# A Stable *Agrobacterium rhizogenes*-Mediated Transformation of Cotton (*Gossypium hirsutum* L.) and Plant Regeneration From Transformed Hairy Root via Embryogenesis

**DOI:** 10.3389/fpls.2020.604255

**Published:** 2020-12-14

**Authors:** Min-Long Cui, Chen Liu, Chun-Lan Piao, Chuan-Liang Liu

**Affiliations:** ^1^College of Agriculture and Food Sciences, Zhejiang A & F University, Hangzhou, China; ^2^College of Bioscience and Biotechnology, Shenyang Agricultural University, Shenyang, China; ^3^Institute of Cotton Research, Chinese Academy of Agricultural Sciences, Anyang, China; ^4^School of Agricultural Sciences, Zhengzhou University, Zhengzhou, China

**Keywords:** cotton (*Gossypium hirsutum* L.), cotyledon, *Agrobacterium rhizogenes*-mediated, transformed hairy root, embryogenesis, plant regeneration, southern blot analysis

## Abstract

Genetic transformation is a powerful tool to study gene function, secondary metabolism pathways, and molecular breeding in crops. Cotton (*Gossypium hirsutum* L.) is one of the most important economic crops in the world. Current cotton transformation methods take at least seven to culture and are labor-intensive and limited to some cultivars. In this study, we first time achieved plantlet regeneration of cotton via embryogenesis from transformed hairy roots. We inoculated the cotyledon explants of a commercial cultivar Zhongmian-24 with *Agrobacterium rhizogenes* strain AR1193, harboring a binary vector pBI-35S::GFP that contained the *NPT II* (neomycin phosphotransferase) gene and the *GFP* (green fluorescent protein) gene as a fluorescent marker in the T-DNA region. 82.6% explants produced adventitious roots, of which 53% showed GFP expression after transformation. 82% of transformed hairy roots produced embryonic calli, 12% of which regenerated into stable transformed cotton plants after 7 months of culture. The integration of *GFP* in the transformed cotton genomes were confirmed by PCR (Polymerase chain reaction) and Southern blot analysis as well as the stable expression of GFP were also detected by semi-quantitative RT-PCR analysis. The resultant transformed plantlets were phenotypically, thus avoiding Ri syndrome. Here we report a stable and reproducible method for *A. rhizogenes*-mediated transformation of *cotton* using cotyledon as explants, which provides a useful and reliable platform for gene function analysis of cotton.

## Introduction

Cotton is an economically important crop well known for providing natural fibers. It also is a producer of seed oil which ranks third in oil production globally, after soybean and canola (Shang et al., 2017). Cotton seed oil is rich in a range of fatty acids, such as oleic acid, stearic acid, and palmitic acid as well as having a low flavor reversion, making it an important oil in the food industry (Liu et al., 2017). In the last century, traditional breeding attempted to improve some agronomic traits of cotton, such as fiber length and quality, disease resistance, and oil yield. However, progress has been unsatisfactory due to the lack of useful genetic resources in cotton. In contrast with classical breeding, genetic transformation is a powerful research tool for use in gene discovery and crop improvement. Recently, many genes have been identified which have putative function in fiber development and the seed oil biosynthesis pathway (Walford et al., 2011; Liu et al., 2017; Shang et al., 2017). Analysis of the recently sequenced genome sheds new light on basic metabolism and further unravels the oil biosynthesis pathway (Du et al., 2018). However, previous cotton transformation protocols have key limitations, including the inefficient regeneration of transformed shoots, with successful transformants obtained through time-consuming and labor intensive methods (Sunilkumar and Rathore, 2001; Leelavathi et al., 2004; Zhao et al., 2006).

Since *Agrobacterium tumefaciens-*mediated transformation and regeneration of cotton from hypocotyls or cotyledons via somatic embryogenesis was first reported by Firoozabady et al. (1987) and Umbeck et al. (1987), other methods of transforming cotton have been described, such as particle bombardment (Finer and McMullen, 1990; John and Keller, 1996; Rajasekran et al., 1996; Liu et al., 2011), utilizing the pollen-tube pathway (Huang et al., 1999; Wang et al., 2004), and a combination of *Agrobacterium* and shoot-apex explants (Zapata et al., 1999). To date, *A. tumefaciens-*mediated transformation has been most widely used for cotton transformation and has introduced some commercially useful genes into cotton cultivars, leading to the subsequent generation of transgenic cotton plants (Leelavathi et al., 2004; Khan et al., 2010; Abdurakhmonov et al., 2014). Whilst eventually successful, these methods are slow, complex selection process, inefficient and also suffer from poor regeneration.

Similar to *A. tumefaciens*, *Agrobacterium rhizogenes* has the ability to transfer T-DNA using the root-inducing (Ri) plasmid to the target plant genome, whilst also inducing the formation of hairy roots (Tepfer, 1984; Seki et al., 2005). *A. rhizogenes* can also transfer T-DNA binary vectors, enabling the production of transformed hairy roots containing genes of interest that are carried on a binary vector (Cho et al., 2000; Cui et al., 2001). Transformed hairy roots are induced rapidly and efficiently from explant tissues and are easy to identify to separate as individual clones. They show rapid growth and have the same genetic characteristics to normal roots. The culture procedure is simple and plants can be maintained for a substantial period of time. Hairy root culture methods have been used in soybean (Kereszt et al., 2007), Medicago (Boisson-Dernier et al., 2001), tomato (Ron et al., 2014), *Saussurea involucrata* (Fu et al., 2005), *Duboisia leichhardtii* (Mano et al., 1989), *Antirrhinum* (Gao et al., 2013). It has also been used for cotton (Triplett et al., 2008; Frankfater et al., 2009; Wubben et al., 2009; Kim et al., 2011; Gao et al., 2013), but whilst coming close, these methods have not tackled the problem of efficient regeneration.

Here, we present an efficient and stable method of transformation mediated by *A. rhizogenes* and for the first-time regeneration of cotton via embryogenesis using the cultivar Zhongmian-24. We show that through production of hairy roots, transformation with a fluorescent marker followed by callus induction, stable transformed plantlets can be regenerated after 6–7 months of culture. This transformation period is shorter compared with other transformation methods and use somatic embryogenesis for efficient regeneration to allow for higher production of stable transformants and providing a reliable tool for the study of gene function in cotton.

### Materials and Equipment

#### Materials

**FIGURE 1:**
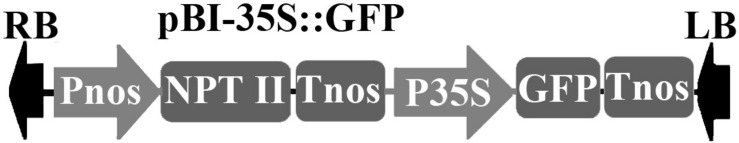
The T-DNA structureof expression vector pBI-35S::GFP. The right border (RB) and left border (LB) of T-DNA were indicated by black arrow. Pnos: Nopaline synthase gene promoter, *NPT* II: The neomycin phosphotransferase II gene, Tnos: Terminator of nopaline synthase gene, P35S: 35S promoter of *cauliflower mosaic virus*, *GFP*: Green fluorescent protein gene.

• Cotton cultivar: Zhongmian-24 (From institute of cotton research of CAAS).
• Agrobacterium strain: AR1193/pBI121-GFP (Figure 1).

#### General Reagents

• Sterile distilled and deionized water.
• Mercury dichloride (HgCl_2_, Shanghai, China).
• Agar (A1296-1, Sigma, Aldrich).
• Gelrite (G1910, Sigma, Aldrich).
• Sucrose (S2792, Sangon Biotech, China).
• MS medium (M519, PhytoTech Labs, United States).
• MSB5 medium (M404, PhytoTech Labs, United States).
• Absolute ethanol (A500737, Sangon Biotech, China).
• Isopropanol (A507048, Sangon Biotech, China).
• RNAiso Plus (9108, Takara, Dalian).
• LB Agar Plate (B530111, Sangon Biotech, China).
• LB sterile liquid medium (B540111, Sangon Biotech, China).
• Yeast Extract (A610961, Sangon Biotech, China).
• Tryptone (A650217, Sangon Biotech, China).
• Sodium chloride (A100241, Sangon Biotech, China).
• Glutamine (816016, Sigma, Aldrich).
• Rifampicin (A600812, Sangon Biotech, China).
• Kanamycin (A600286, Sangon Biotech, China).
• 1-Naphthaleneacetic acid (NAA) (N0640, Sigma, Aldrich).
• 3-Indole acetic acid (IAA) (A600723, Sangon Biotech, China).
• 2.4-Dichlorophenoxyacetic acid (2.4-D) (D7299, Sigma, Aldrich).
• Kinetin (KT) (K0753, Sigma, Aldrich).
• Acetosyringone (AB1111-Sangon Biotech, China).
• SV total RNA isolation system (Z3101, Promega).
• Super Script III First-strand synthesis Kit (18080, Invitrogen).
• PCR Dig Probe Synthesis Kit (11636090910, Roche).
• DIG High Prime DNA Labeling and Detection Starter Kit I (11745832910, Roche).
• Molecular-Weight Marker II (11218590910, Roche).
• cefotaxime (A601276, Sangon Biotech, China).

#### Equipment and Materials

• Clean bench (Boxun, China).
• Shaker (THZ-C-1, Taicang, China).
• Fluorescence microscope (Leica165 FC) (Leica, Germany).
• GeneAmp PCR System 2700 (ABI, United States).
• 20 ml Syringes.
• Plastic pot (5 cm × 5 cm × 12 cm).
• Plastic sterile Petri dishes (90 mm ×17 mm).
• 50 ml conical polypropylene tubes.
• Scalpel (no. 23).
• 15–20 cm Forceps.
• Syringe filter (0.22 μm).

#### Reagent Setup

• *1 mg/ml IAA*: Add 1 N HCl dropwise to 50 mg IAA until completely dissolved. Make up to 50 ml with distilled water, store at 4°C.
• *1 mg/ml NAA*: Add 1 N HCl dropwise to 50 mg NAA until completely dissolved. Make up to 50 ml with distilled water, store at 4°C.
• *1 mg/ml 2,4-D*: Add 1 N NaOH dropwise to 50 mg 2,4-D until completely dissolved. Make up to 50 ml with distilled water, store at 4°C.
• *1 mg/ml Kinetin (KT)*: Add 1 N NaOH dropwise to 50 mg kinetin until completely dissolved. Make up to 50 ml with distilled water, store at 4°C.
• *20 mg/ml acetosyringone stock*: Dissolve 1 g acetosyringone in 50 ml of dimethyl sulfoxide and store in the dark at −20°C.
• *Liquid LB medium* (10 g tryptone, 5 g yeast extract, 10 g NaCl per liter, adjusted to pH 7.2): Once sterilized by autoclaving, store at 4°C for several months.
• *Plant tissue culture media*.

The media used in this study were indicated Table 1.

**TABLE 1:** Composition of the media used in this study.

Medium	Composition
MS: seed germination	Macro and micro salts of MS +1x vitamins of MS + 30 mg/L sucrose + 3.5 g/L gelrite, pH 5.8
CM: co-cultivation	Macro and micro salts of MS +1x vitamins of MS + 30 mg/L sucrose + 0.2 mg/L NAA + 40 mg/L acetosyringone + 3.5 g/L gelrite, pH 5.5
RIM: hairy root induction	Macro and micro salts of MS +1x vitamins of MS + 30 mg/L Sucrose + 0.2 mg/L NAA + 200 mg/L cefotaxime + 3.5 g/L gelrite, pH 5.8
PCIM: primary callus induction	Macro and micro salts of MS +1x vitamins of MS + 30 mg/L sucrose + 0.1 mg/L IAA + 0.15 mg/L 2. 4-D + 0.2 mg/L KT + 200 mg/L cefotaxime + 3.5 g/L gelrite, pH 5.8
ECIM: embryogenic callus induction	Macro and micro salts of MS +1x vitamins of MS + 30 mg/L sucrose + 0.01 mg/L IAA + 0.01 mg/L 2. 4-D + 0.02 mg/L KT + 50 mg/L asparagine + 75 mg/L glutamine + 200 mg/L cefotaxime + 3.5 g/L gelrite, pH 5.8
SEIM: somatic embryo induction	Macro and micro salts of MS +1x vitamins of B5 + 30 mg/L sucrose + 50 mg/L asparagine + 75 mg/L glutamine + 50 mg/L cefotaxime + 3.5 g/L gelrite, pH 5.8
SEGM: somatic embryos germination	Macro and micro salts of MS + 1x vitamins of B5 + 30 mg/L sucrose + 50 mg/L cefotaxime + 3.5 g/L gelrite, pH 5.8

*MS, Murashige and Skoog medium; B5, Gamborg B5-vitamin (Gamborg et al., 1968); NAA, 1-Naphthaleneacetic acid; IAA, Indole-3-acetic acid; 2.4-D, 2, 4-Dichlorophenoxyacetic acid; KT, Kinetin.*

## Methods

### Plant Material

(1) The cultivar Zhongmian-24 was used in this study.
(2) The seeds from the removed coat were sterilized with 0.1% HgCl_2_ for 5 min and rinsed five times with sterile distilled water for 3 min to remove the disinfectant completely.
(3) The seeds were germinated on MS medium (Murashige and Skoog, 1962) supplemented with 3% sucrose and 3.5% phytagel and the pH was adjusted to 5.8 in plastic pots.
(4) The plant materials were grown at 25°C under a 16/8 h light/dark photoperiod at an intensity of 60 μmol m^–2^ s^–1^. After 2 weeks of culture.
(5) Fully expanded cotyledons that were green in color were used for the transformation experiment.

### Transformation Vector and *Agrobacterium* Strain

(1) The *A. rhizogenes* strain AR1193 harboring the binary vector pBI-35S::GFP (Figure 1) was used for the transformation of cotton.
(2) The plasmid pBI-35S::GFP containing the *NPT II* gene and *GFP* gene in the T-DNA region. The pBI-35S::GFP expression vector was introduced to *A*. *rhizogenes* AR1193 cells by electroporation method (Shen and Forde, 1989).

### Transformation and Hairy Root Induction

(1) Pick up a little of *A. rhizogenes* strain AR 1193/pBI-35S::GFP of stocked at −80°C was grown in 5 mL of LB liquid medium added 50 mg/L kanamycin (used 50 ml conical tube), at 28°C, 200 rpm, for 20–24 h.
(2) Take 1 ml, transfer to a sterile 50 ml conical tube containing 40 ml liquid CM medium (without gelrite) (Table 1), about OD 0.1–0.2 used to infection of cotyledon of cotton.
(3) The 2-week-old green and expanded cotyledons were excised from the seedlings and wounded with a sterile scalpel, infection with the diluted *Agrobacterium rizhogenes* AR 1193/pBI-35S::GFP suspension for 8 min.
(4) The infected cotyledons transferred to CM solid medium (Table 1) and co-culture for 48 h at 20°C in the dark condition.
(5) The cotyledons were transferred to RIM (Table 1) and hairy roots were induced. The infection experiment was repeated three times.

### Embryonic Induction and Regeneration of Plantlets From Transformed Hairy Roots

(1) Hairy roots (4–5 cm) were excised from the cotyledons, cut into ∼1–2 cm segments, and placed on PCIM (Table 1) to induce primary calli.
(2) The hairy root segments or calli were changed to the same fresh medium at 3-week intervals.
(3) After ∼8 weeks of culture, the induced calli were transferred to ECIM (Table 1) to induce embryonic calli.
(4) After culturing for 2 months, the induced embryogenic calli were transferred to SEIM (Table 1) to induce somatic embryos.
(5) The induced embryos were transferred to SEGM (Table 1) to allow elongation and the development of normal plantlets.
(6) The normal like plantlets were transferred to MS medium containing 250 mg L^–1^ cefotaxime induce roots.
(7) Selected, normal-like plantlets were transferred to nutrient soil and grown in the culture room at 25°C, 16 h light/8 h dark condition.

### Observation of GFP Expression

Green fluorescent protein activity was observed in emerging hairy roots and induced primary calli, and at embryogenic calli induction, somatic embryo induction, and rooting stages, using a fluorescence microscope (Leica165 FC) equipped with a GFP2 filter. The images were captured using an imaging system DFC310 FX (Leica) and the Leica software (LAS V4.2). Digital image processing was performed using Adobe Photoshop CS2.

### Detection of *GFP* Gene in Transformed Calli and Plantlets by PCR

(1) Genomic DNA was extracted from fresh embryo calli and young leaves of geminated somatic embryos using the CTAB method (Rogers and Bendich, 1985).
(2) Detection of the *GFP* gene was used following primer sets: GFP(+) ATGGTGAGCAAGGGCGAG GAGC and GFP(−) TTACTTGTACAGCTCGTCCATGC.
(3) Reactions were carried out on a GeneAmp PCR System 2700, and the PCR program was set with denaturation at 94°C for 5 min followed by 35 cycles at of 94°C for 30 s, 57°C for 30 s, and 72°C for 40 s, and a final extension at 72°C for 5 min.
(4) The PCR products were separated use 1% agarose gels.

### Semi-Quantitative RT-PCR Analysis of GFP Expression

(1) Total RNA was extracted from 100 mg young leaves of a wild-type and nine transformed cotton plantlets combination with SV total RNA isolation kit and RNase-free DNase (Promega), respectively.
(2) The first-cDNA was synthesized using Super Script III kit (Invitrogen).
(3) The GFP primer set: GFP(+) ATGGTGAGCAAGG GCGA GGAGC and GFP(−)TTACTTGTACA GCTCGTCCATGC, and a set of cotton ubiquitin gene primer: Ubi (+) GAAGGCATTCCACC TGACCAAC and Ubi(−) CTTGACCTTC TTCTTCTTGTGCTTG as a positive control were used RT-PCR analysis.
(4) Semi-quantitative RT-PCR were carried out according to described by Gao et al. (2015).
(5) The PCR products were analyzed 1% agarose gels and confirmed by sequencing.
(6) The electrophoretogram processing was performed with Adobe Photoshop 7.0.

### Detection of Transformed Plantlets by Southern Blot Analysis

To confirm the stable integration of transgenes was performed Southern blot analysis.

(1) Total DNA was isolated from young leaves geminated from somatic embryos of seven PCR positive cotton plantlets and a wild type cotton plant using the CTAB extraction method (Rogers and Bendich, 1985).
(2) 20 μg total genomic DNA were completely digested with *Hind* III, separated by electrophoresis on 1% agarose gel at 200 V for 10 h, and then transferred onto a Hybond-N+ membrane (Amersham).
(3) The membrane was hybridized a digoxin-labeled full-length *GFP* gene probe, which synthesized by a PCR Dig Probe Synthesis Kit (Roche) using the primer set above.
(4) The hybridization was performed at 60°C using the protocol provided for the DIG High Prime DNA Labeling and Detection Starter Kit I (Roche).
(5) All hybridization and signal detection procedures were carried out according to the manufacturer’s instructions (Roche).

## Results

### Hairy Root Induction From Cotyledon Explants

**FIGURE 2:**
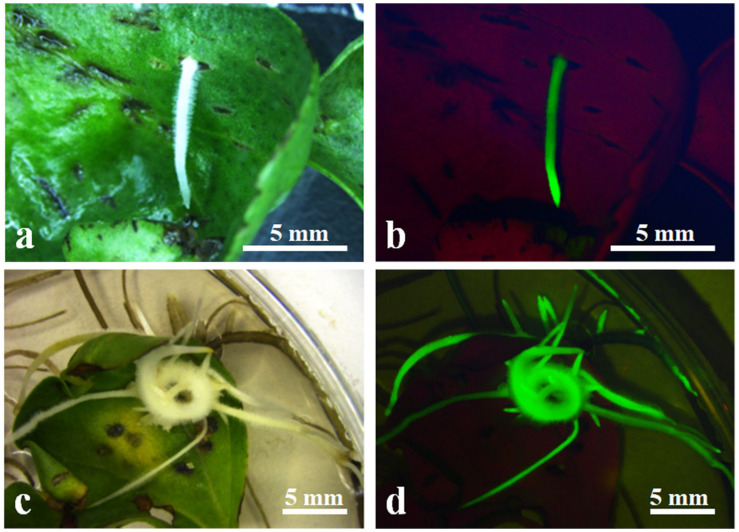
Induction of transformed hairy roots from wounded 2-week-old cotyledon. **(a)** Emerging hairy roots from wounded cotyledon after 10 days of infection *A. rhizogenes* AR1193/pBI-35S::GFP; **(b)** GFP expression on induced hairy root in **(a)**; **(c)** induced hairy root from wounded cotyledon after 4 weeks of infection *A. rhizogenes* AR1193; **(d)** GFP expression on induced hairy roots in **(c)**.

Hairy roots induced by *A. rhizogenes* are a visible and simple marker that permits the selection of transformed roots. To establish stable conditions for the production of cotton hairy roots, a combination of the *A. rhizogenes* strain AR 1193/pBI-35S::GFP and 2-week-old hypocotyl segments, leaf explants, and cotyledon of cotton were examined. After 2 weeks of infection, adventitious roots emerged from wounded cotyledons, which were growing fast on RIM (Figures 2a,c). Emerging adventitious roots were also observed from explants of infected hypocotyl segments and leaves, but the number of adventitious roots was lower than the number of cotyledons (data not shown). Therefore, cotton transformation was performed using a combination of *A. rhizogenes* AR1193/pBI121-GFP and 2-week-old cotyledons and the experiment was repeated three times. After 4 weeks of infection, 79.6%, 83%, and 85% of the infected cotyledons emerged with a large number of adventitious roots, obtained 229, 346, and 93 independent adventitious roots, respectively; among them, 57.6%, 53.7%, and 47.3% of adventitious roots showed strong GFP expression, respectively (Table 2 and Figures 2b,d).

**TABLE 2:** Induction of hairy roots from cotyledons of Zhongmian-24, after 4-weeks infection with *A. rhizogenes* AR1193 containing a binary vector pBI-35S::GFP on RIM.

Number of cotyledon explants^a^	Number of emerged adventitious roots cotyledon explants	Number of independent adventitious roots	Number of GFP-positive roots	Frequency of GFP-positive roots (%)^b^
177	141(79.66%)	229	132	57.64
124	103(83.06%)	346	186	53.76
142	121(85.21%)	93	44	47.31

*^*a*^Infection experiment was repeated three times. ^*b*^(Number of GFP-positive roots/number of adventitious roots) × 100%.*

### Induction of Embryos From Transformed Hairy Roots

**FIGURE 3:**
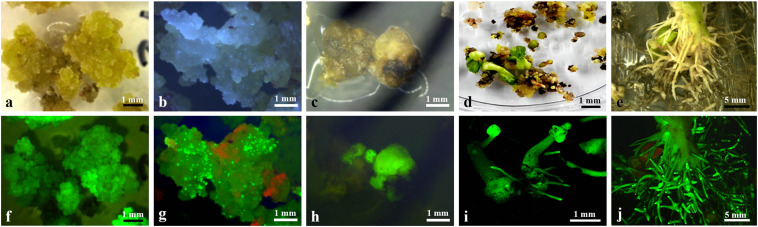
Callus induction and plantlet Regeneration from transformed hairy roots. **(a)** Induction of primary callus from a transformed hairy root after 2 months culture on PCIM; **(b)** Induction of an embryogenic callus after 2 months culture on ECIM; **(c)** Appearance of embryos after 4 weeks culture on SEIM; **(d)** Green shoots germination from somatic embryos after 2 weeks culture on SEGM; **(e)** Numerous abnormal roots development from a transformed cotton plantlet; **(f)** GFP expression in induced primary callus **(a)**; **(g)** GFP expression in induced embryogenic callus **(b)**; **(h)** GFP expression on globular or oval-like embryos **(c)**; **(i)** GFP expression on germinated plantlets **(d)**; **(j)** GFP expression the abnormal roots of a transformed cotton plantlet. Scale bars were indicated.

Choice independent hairy roots with showing strong GFP expression and fast growing were cut into pieces of length 1–2 cm and placed on PCIM-induced calli. Three-to-four weeks after the culture, yellowish and soft calli were observed as emerging, mainly from the cut end, and after 2 months of culture, about 82% of the hairy root segments produced yellowish and soft calli at the cut end and inner side (Figures 3a,f). The yellowish and soft calli were transferred to ECIM-induced embryos. After two subcultures on ECIM (changing the medium every 3 weeks), newly formed creamy-white and friable calli or a few green globular embryos appeared on their surface, and the green globular embryos exhibited strong GFP expression (Figures 3b,g). The creamy-white and friable calli were transferred to SEIM-induced mature somatic embryos. Following 2 months of culture on SEIM medium, numerous globular or oval-like embryos developed (Figure 3c,h).

### Plantlet Regeneration

The green globular or oval-like embryos were transferred to SEGM. The embryos rapidly developed a green shoot and primary root (Figure 3d), which showed strong GFP expression (Figure 3i). After 4 weeks of culture, plantlets were obtained from 23 independent hairy root lines, and the regenerated plantlets were normally grown on MS medium containing 50 mg/L kanamycin. Among them, a few regenerated plantlets showed severe Ri syndrome such emerged numerous adventitious roots (Figures 3e,j), and some regenerated plantlets no showed clear morphological alteration in the roots and leaves (data not shown) which morphologically similar to the non-transformed cotton plant.

### Molecular Analysis of Transformed Cotton Plantlets

**FIGURE 4:**
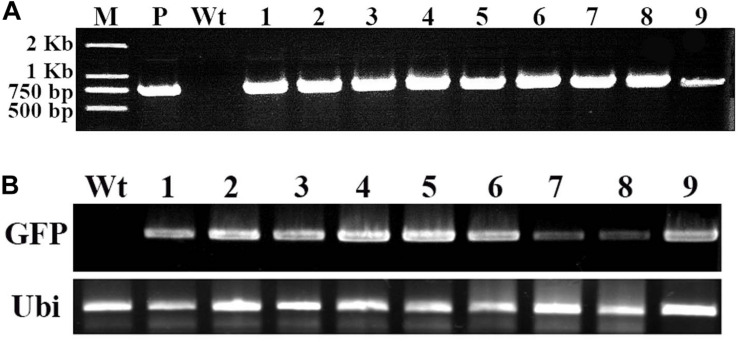
Molecular analysis of transformed cotton plantlets. Total RNA and DNA were extracted in young leaf of a wild-type cotton plant and nine independent transformed plantlets, which showing stable GFP expression. **(A)** The PCR analysis for detection of *GFP* in transformed cotton plantlets. M, 2-kb DNA ladder marker; P, pBI-35S::GFP plasmid DNA (positive control); Wt, wild-type cotton DNA (negative control); Lane 1–9, nine independent transformed cotton plantlets; **(B)** The RT-PCR analysis for GFP expression in transformed cotton plantlets. The GFP-specific primers and ubiquitin-specific primers of cotton were used. Wt, wild-type plant; lanes 1–9, independent transformed plantlets of cotton.

To confirm the *GFP* gene in the regenerated putative transgenes cotton plantlet genomes, DNA was isolated from nine independent cotton plantlets that showing stable GFP expression and a non-transformed cotton plant, and using *GFP* specific primers were performed PCR analysis. The result, the 780-bp *GFP* specific bands were detected in each of the nine selected cotton plants, however, the band was not amplified in the non-transformed cotton plant (Figure 4A). Next to detection of the GFP expression in the transgenes cotton plantlets, semi-quantitative RT-PCR was performed using a GFP specific primer pairs. The results of GFP was transcribed and expressed in all nine tested cotton plantlets, but no expression of *GFP* was detected in non-transformed wild-type cotton plant (Figure 4B).

### Southern Blot Hybridization Analysis

**FIGURE 5:**
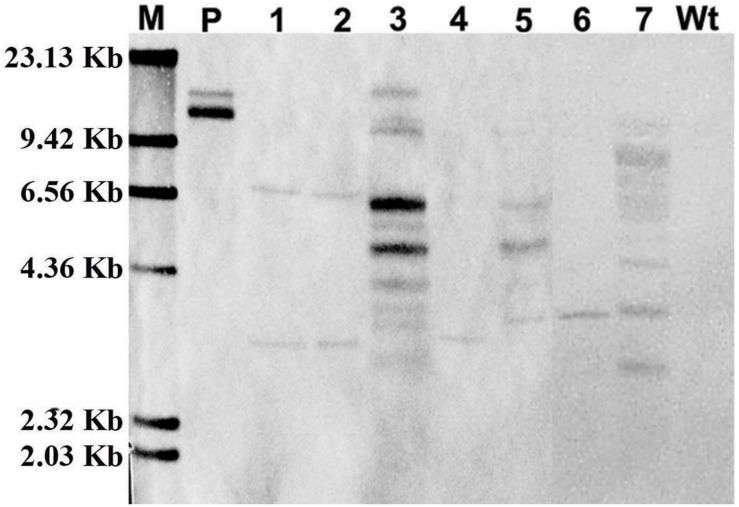
Confirmation of *GFP* integration in transformed cotton plants by Southern blot analysis. The genome DNA was extracted from a wild type cotton plant and independent seven transformed cotton plants. The Southern blot analysis were used 20 μg genome DNA of digested with *Hind* III, respectively, and hybridized with a DIG labeled 720-bp *GFP* coding fragment amplified by PCR. M, DIG labeled DNA marker (Roche); P, pBI-35S::GFP plasmid; Lane 1–7, The DNA from seven independent transformed cotton plants, respectively; Wt, The DNA from a wild type cotton plant.

Furthermore, to confirm the stable integration of *GFP* in the cotton plantlet genomes, genomic DNA from seven independent GFP positive plantlets were carried out Southern blot analysis using Dig-labeled 780 bp GFP fragment as a probe (Figure 4A). The results all seven independent plants were displayed different patterns. Among them, two plant lets (line 4 and 6) were showed one copies, two plant lets (line 1 and 2) were showed two copies, and three plant lets (line 3, 5, and 7) were showed multi copies. However, no hybridization signal was detected in non-transformed wild type cotton plant (Figure 5).

### Summary of Transformation Process

**FIGURE 6:**
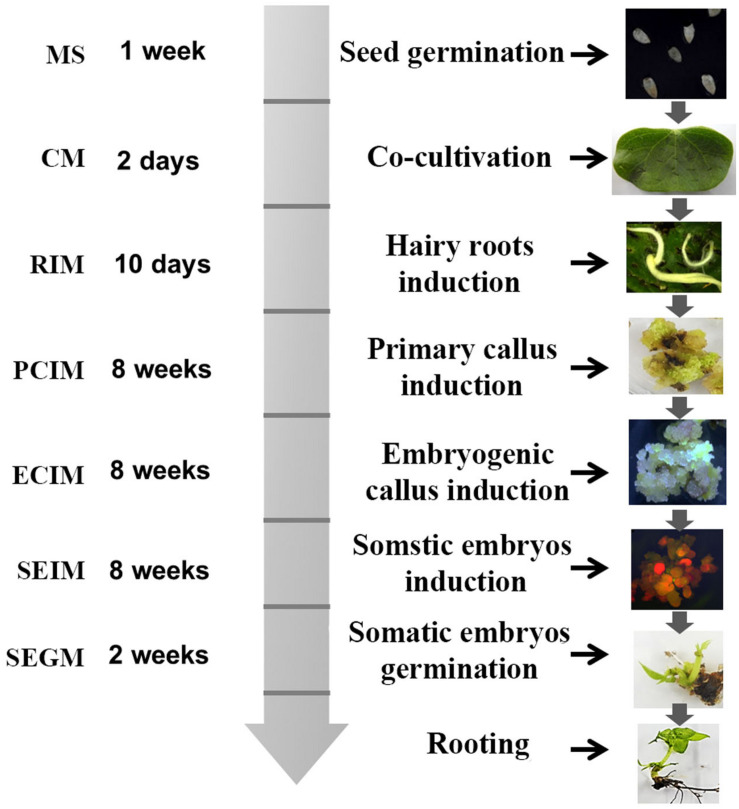
Diagram of *A. rhizogenes-*mediated transformation of *cotton*. The time schedule and medium are indicated on the left and the corresponding figures were showed on the right.

The method for transformation of cotton by *A. rhizogene*s AR1193 is summarized in Figure 6. Two-week-old cotyledons were inoculated with *A. rhizogenes* strain AR1193, and after 2 days of co-cultivation, the cotyledons were transferred onto hairy root induction medium (RIM). The induced hairy roots were subsequently cut into 1–2 cm pieces for culturing on PCIM. After 2 months, the induced calli were transferred to ECIM to induce embryogenic calli. After a further 2 months, the embryogenic calli were transferred to SEIM. At the end of next 2 months, the embryos were transferred to the germination medium (SEGM) to produce plantlets. The plantlets were then transferred to a rooting medium to induce roots and were subsequently potted in soil.

## Discussion

We developed an *A. rhizogene*s-mediated stable transformation method for a commercial cotton cultivar Zhongmian-24, and the first time successfully regenerated plants from transformed hairy roots via somatic embryogenesis. Expression of the *GFP* reporter gene was detected at each stage from inoculation to plantlet development, and the *GFP* gene integration was also confirmed by both PCR and Southern blot analysis. The period from induction of hairy roots to mature plant development needed is about 7 months, with a transformation efficiency of 12%. These results show that this transformation method is a simple and suitable for selection transformed hairy roots and obtain transformed plants as well as a useful and reliable platform for gene function analysis of cotton.

In plant transformation process widely use the kanamycin-, hygromycin- or bialaphos (bar) resistant genes as selectable markers (Manoharan and Dahleen, 2002; Ceasar and Ignacimuthu, 2011; Acanda et al., 2017). However, these antibiotics or herbicides were retard the plant cell differentiation and adventitious shoots formation during tissue culture process (Catlin, 1990). The *A. rhizogenes*-mediated transformation compare to *A. tumefaciens*-mediated transformation has some advantages such as induce transformed hairy root rapidly and efficiently from explant, easy to identify individual transformed clones and no requires selection marker genes, therefore this technique has also been used for study gene functions, plant transformation and study of secondary metabolism (Cui et al., 2001; Gao et al., 2013; Aggarwal et al., 2018; Ho-Plágaro et al., 2018). In this study, *A. rhizogenes* strain 1193 was used, and the characters of hairy roots that fast elongation and increase lateral roots on non-plant hormone media as selection marker, 342 independent hairy root clones were obtained after 4 weeks infection (Table 2). Among them 79 independent hairy roots were emerged embryogenic callus and 54 hairy roots were obtained transformed plant lets. In this study, the *GFP* also used as a selection marker, and monitored the *GFP* expression at different developmental stages as well as detected that *GFP* expression from hairy root induction to development of plantlets on transformed calli (Figure 3). In this study, we also found that about 50% adventitious roots were no showed visible GFP expression, among them some adventitious roots showed Ri syndrome such as fast elongation and increase blanch, however, other some adventitious roots that are probably non-transformed escape root were stopped elongation and died early (data not shown). Therefore, this two-step selection method is more reliable and fast obtain transformed calli than those reported previously.

Establishment of a stable and efficient transformation method improves the amount of regenerable explants. In cotton, the hypocotyl, cotyledon, the shoot apex, and embryogenic calli have been used as materials for transformation (Thomas et al., 1995; Rajasekran et al., 1996; Zapata et al., 1999; Leelavathi et al., 2004; Wu et al., 2008; Jiang et al., 2012). Several studies have reported that cotyledon explants produce more hairy roots than hypocotyl segments in cotton and other plants, when inoculated with *A. rhizogenes* strains (Xu et al., 2004; Triplett et al., 2008). In this study, we also used the cotyledon and hypocotyl as material for inoculation with *A. rhizogene*s strain AR1193 and observed similar results. We found that even though both cotyledon and hypocotyl segments can be infected by AR1193, the cotyledon is more reliable. Numerous adventitious roots appeared on the surface of each cotyledon after 10 days of infection (Figure 2a), which was faster than hypocotyl segments which took 15 days (data not shown). 57% of the independent adventitious roots exhibited strong green fluorescence (Figures 2b,d). 82% of the hairy roots showing strong GFP expression were derived from cotyledons, which produced yellowish and soft primary calli in the presence of low concentrations of 2.4-D (0.1 mg/L) in 2 months of culture (Figure 3a). Among these, 22% produced embryogenic calli within 2 months of culture, and ultimately 12% of the independent hairy roots yielded normal-like transformed plantlets after 7 months of culture (Table 3). These results show that the cotyledon is the best material for *A. rhizogenes -*mediated transformation of cotton, and that the cotyledon explants of cultivar Zhongmian-24 and the *A. rhizogenes* strain AR 1193 is a suitable combination for cotton transformation.

**TABLE 3:** Frequency of *A. rhizogenes*-mediated cotton transformation via an embryogenic process.

No. of GFP Positive independent roots	No. of growing primary callus roots^a^	No. of growing embryogenic callus roots^b^	No. of regenerated plantlet roots (%)^c^
132	109 (82.58%)	33	17 (12.88%)
186	155 (83.33%)	37	22 (11.83%)
44	35 (79.55%)	9	5 (11.36%)

*^*a*^Hairy roots cut into 1–2 cm segments were cultured on PCIM for 2 months.*

*^*b*^The primary callus transferred on SEIM was cultured for 2 months. ^*c*^(Number of regenerated plantlets/number of GFP-positive independent roots) × 100%.*

*Agrobacterium rhizogenes* harbors the *Ri* plasmid, which infects through wounded plant tissue induce hairy roots (Tepfer, 1990; Christey, 2001). The *rol* genes modify the hormone levels of the host plants for proliferation of hairy roots. Plants regenerated from hairy roots can show altered phenotypes, known as Ri syndrome, such as dwarfism, reduced apical dominance in both stem and roots, wrinkled leaves, and altered flower morphology (Cui et al., 2001; Jiang et al., 2012). In present study, we found that supplemented 0.2 mg/L NAA auxin in the hairy root induction medium (RIM) strongly promoted hairy root formation on cotyledon explants and adventitious root elongation. This result was in contrast to the findings of Triplett et al. (2008) who reported that in cotton cotyledon explants inoculated with a highly virulent strain of *A. rhizogenes* 135834, the best condition for hairy root induction was the use of hormone-free MS medium, and that supplementation with low concentrations of auxin reduced hairy root production and affected the hairy root growth. We also found that some transformed cotton plants showed normal like morphology at the apical meristems and leaves, and no obvious severe Ri syndrome was observed in them (data not shown). Although we could not confirm the expression patterns of *rol* genes in the transformed cotton plants in this study, we postulate that the normal-like phenotype of transformed cotton plants and the low concentrations of supplemented auxin promoted hairy root formation on cotyledon could be a result of *A. rhizogenes* AR1193 being a low-virulence strain; therefore, the levels at which the *rol* genes synthesized hormones might not have disturbed the internal cytokinin-auxin balance in the cells and tissues, nor the organ development process in host cotton plants. Taken together, our results suggest that the low-virulence *A. rhizogenes* AR1193 is a suitable strain and a combination with low concentration of auxin is a best condition for *A. rhizogenes-*mediated cotton transformation.

Through present study, we first time reports for the development of an efficient and reliable cotton plantlets regeneration method mediated by *A. rhizogenes*. This method of *A. rhizogen*es-mediated transformation has several advantages. First, hairy roots act as a visible marker for the selection of transformed roots, which makes the process simple and fast, thus reducing the selection period of transformed calli. Second, regenerated transformants can be obtained 6–7 months after *A. rhizogen*es infection via embryogenesis. Furthermore, the transformation efficiency was found to be 12% of cotton plants with a normal phenotype. Therefore, we provide a useful tool for use in the functional analysis of cotton genes and with potential application to the molecular breeding and genome editing of cotton.

## Data Availability Statement

The original contributions presented in the study are included in the article/supplementary material, further inquiries can be directed to the corresponding author.

## Author Contributions

M-LC designed the experiments. M-LC and CL carried out the experimental work. C-LP participated the transformation and tissue culture. M-LC, CL, and C-LL wrote the manuscript. All authors read and approved the final manuscript.

## Conflict of Interest

The authors declare that the research was conducted in the absence of any commercial or financial relationships that could be construed as a potential conflict of interest.

## Abbreviations

MS: Murashige and Skoog medium
B5: Gamborg B5-medium
*GFP*: green fluorescent protein gene
KT: kinetin
NAA: 1-Naphthaleneacetic acid
IAA: Indole-3-acetic acid
2.4-D, 2: 4-Dichlorophenoxyacetic acid
*NPT II*: neomycin phosphotransferase gene
CTAB: hexadecyltrimethylammonium bromide
PCR: Polymerase Chain Reaction.
